# Relative contribution of lateral vestibular neuron and abducens internuclear neuron inputs to the discharge activity of medial rectus motoneurons

**DOI:** 10.1007/s00429-023-02736-6

**Published:** 2023-11-30

**Authors:** Rosendo G. Hernández, Beatriz Benítez-Temiño, Rosa R. de la Cruz, Angel M. Pastor

**Affiliations:** Departamento de Fisiología, Facultad de Biología, Universidad de Sevilla, 41012 Sevilla, Spain

**Keywords:** Eye movements, Vestibulo-ocular reflex, Oculomotor system, Medial longitudinal fascicle, Syndrome of internuclear ophthalmoplegia, Extracellular single-unit recordings, Lateral vestibular nucleus

## Abstract

Medial rectus motoneurons mediate nasally directed horizontal eye movements. These motoneurons receive two major excitatory inputs, from the abducens internuclear neurons (ABD Ints) and neurons of the lateral vestibular nucleus whose axons course through the ascending tract of Deiters (ATD). In the present work, we have recorded in the alert chronic cat preparation the discharge activity of these two premotor neurons simultaneously with eye movements, to discern their relative contribution to the firing pattern of medial rectus motoneurons. ABD Int discharge was accurately correlated with eye movements, displaying high sensitivities to eye position and eye velocity. ATD neurons also discharged in relation to spontaneous and vestibular eye movements but showed significantly lower eye position and eye velocity sensitivities. Outstandingly, ATD neurons presented a significantly lower eye position threshold for recruitment compared to both ABD Ints and medial rectus motoneurons. Therefore, ATD neurons exhibited eye position and velocity signals during spontaneous and vestibular eye movements, which were of lower magnitude than those of ABD Ints, but due to their low recruitment threshold, they could play a significant role in facilitating ABD Int signal transmission onto medial rectus motoneurons.

## Introduction

Medial rectus motoneurons are responsible for generating the adducting movement of the ipsilateral eye, i.e., in the nasal direction. These motoneurons receive two principal inputs from pontine structures, both of which are of excitatory nature (Büttner-Ennever 2006; Carpenter and Carleton 1983). First, neurons of the ventrolateral vestibular nucleus project ipsilaterally through the ascending tract of Deiters (ATD) towards medial rectus motoneurons. ATD axons course through the pontine reticular formation between the brachium conjunctivum and the medial longitudinal fascicle (MLF) (Markham et al. 1986; Reisine and Highstein 1979; Reisine et al. 1981). They are second-order vestibular neurons that receive disynaptic inputs from the horizontal semicircular canals (Baker and Highstein 1978). The second group of pontine afferent is represented by the abducens internuclear neurons (ABD Ints), which lay intermingled with the motoneurons within the abducens nucleus. Their axons cross the midline and course through the contralateral MLF to target medial rectus motoneurons (Bienfang 1978; Delgado-Garcia et al. 1986). By means of recording (Delgado-Garcia et al. 1986; Fuchs et al. 1988) and lesion experiments (de la Cruz et al. 2000; Evinger et al. 1977; Gamlin et al. 1989; Lee et al. 2022), ABD Ints have been demonstrated to be responsible for the conjugation of horizontal eye movements. Unilateral MLF injury impairs the ipsilateral eye to adduct beyond the midline, and consequently, leads to the failure of this eye to cross beyond the central gaze position into the contralateral oculomotor hemifield (de la Cruz et al. 2000). These oculomotor deficits caused by MLF lesion are known in clinics as the syndrome of internuclear ophthalmoplegia (Carpenter and McMasters 1963; Christoff et al. 1960; Fiester et al. 2020; Pola and Robinson 1976; Virgo and Plant 2017), which disturbs the execution of versional horizontal eye movements.

ABD Ints have been previously recorded in the alert behaving animal and their discharge pattern was characterized as tonic-phasic (de la Cruz et al. 2000; Delgado-García et al. 1986; Fuchs et al. 1988). Their firing frequency is related to eye position (tonic component) and eye velocity (phasic component), thus displaying eye position and eye velocity sensitivities, for spontaneous and vestibularly induced eye movements. Medial rectus motoneurons also display a tonic-phasic firing pattern similar to that of their afferent ABD Ints (de la Cruz et al. 1989). By contrast, little is known about the firing characteristics of ATD neurons, and only one work carried out under alert conditions describes that these neurons encode head velocity and a weak eye position signal, but a quantification of the signals displayed by these afferents is missing (Reisine et al. 1981). The fact that ABD Ints provide medial rectus motoneurons with all eye movement-related signals makes it difficult to understand the role of the ATD input, which has even been suggested to be superfluous (Pola and Robinson 1978).

On the other hand, intracellular electrophysiological experiments have revealed that VIIIth nerve stimulation generates in medial rectus motoneurons excitatory postsynaptic potentials (EPSPs) of large amplitude. These EPSPs reverse polarity with less current than those produced (monosynaptically) following the electrical stimulation to the ABD Int. The conclusion is that ATD synaptic boutons are located closer to the soma of medial rectus motoneurons than those arriving from the ABD Int pathway (Baker and Highstein 1978; Highstein and Baker 1978). Electron microscopy studies have confirmed that indeed ATD terminals contact medial rectus motoneurons preferentially on the soma and proximal dendrites, whereas ABD Int synaptic endings terminate mainly on distal dendrites (Markham et al. 1986; Nguyen et al. 1999).

The present work aims to investigate and compare the signals displayed by these two major inputs converging on medial rectus motoneurons to shed light on the distinctive role they play in the information processing carried out by the medial rectus motoneurons, as the final common pathway for oculomotor behavior. For this purpose, we have used the chronic alert animal preparation. Therefore, we have analyzed and compared the firing rate of ATD neurons, ABD Ints, and that of their postsynaptic medial rectus motoneurons, with respect to eye position and velocity, during eye movements of spontaneous and vestibular origin.

## Materials and methods

### Surgical procedures

Female cats weighing 2.0–2.5 kg were used (Universidad de Córdoba, Spain). Experimental procedures followed European (2010/63/EU) and national legislation (R.D. 53/2013, BOE 34/11370-421) and were ethically approved (P10-CVI-6053). Care was taken to reduce the number of animals used and to refine experimentation.

Four animals were used for the recordings of ATD and ABD Ints under alert conditions. Medial rectus motoneuron recordings were obtained from our previous publication (Hernández et al. 2017).

**Fig. 1:**
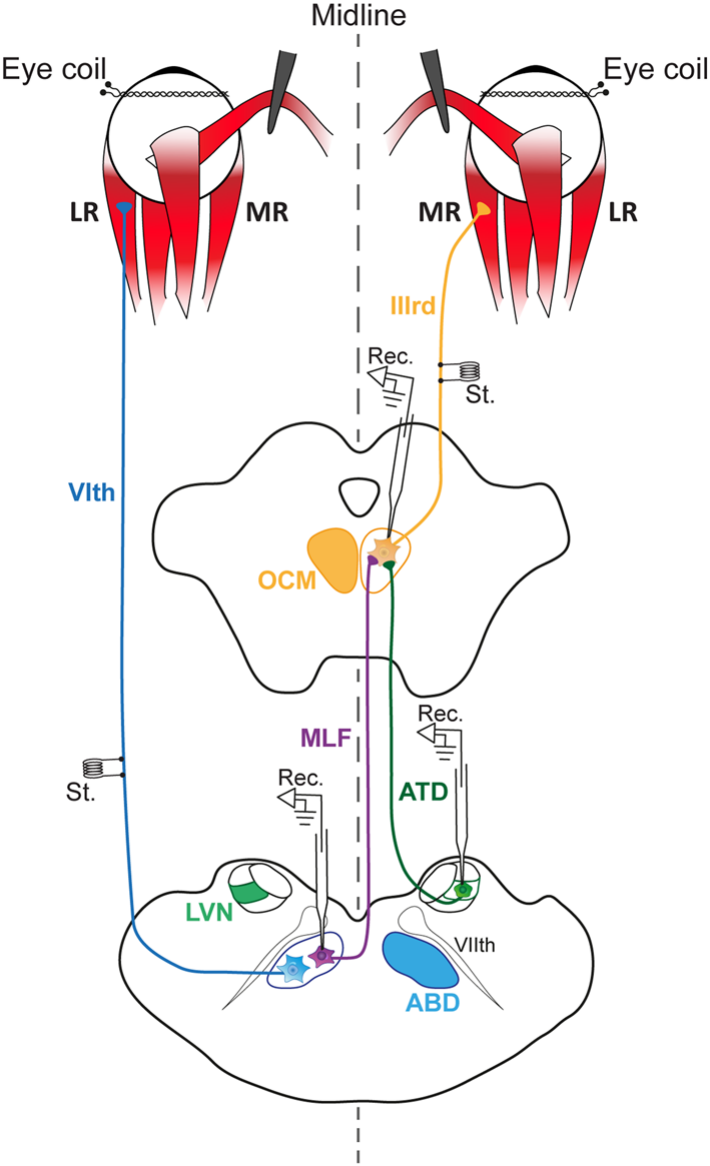
Schematic diagram of the experimental design. Extracellular single-unit recordings (Rec.) were carried out in medial rectus (MR) motoneurons (in orange) of the oculomotor nucleus (OCM), the contralateral abducens (ABD) internuclear neurons (in purple), and the ipsilateral lateral vestibular neurons (LVN, in green). Axons of these two afferents course through the medial longitudinal fascicle (MLF) or by the ascending tract of Deiters (ATD), respectively. Eye coils were implanted bilaterally to record ocular movements. A bipolar stimulating electrode (St.) was implanted in the IIIrd or VIth nerve for the electrophysiological activation of motoneurons. LR stands for lateral rectus muscle, which is innervated by the abducens motoneurons (in blue). VIIth stands for facial nerve

Animals were instrumented for chronic recordings as previously described (Calvo et al. 2022). Briefly, after a vagolytic injection of atropine sulfate (0.5 mg/kg, i.m.), animals were anesthetized with a mixture of ketamine hydrochloride (20 mg/kg, i.m.) and xylazine (1 mg/kg, i.m.). Stereotaxic surgery was required to implant stimulating electrodes. Scleral coils sutured to the sclera of each eye and a recording chamber in the supraoccipital bone were also implanted in the same procedure. Two bipolar electrodes, made of 125 µm silver insulated wire, were implanted at the exit from the brainstem of the VIth nerves for stimulation purposes (Fig. 1, St.; only the left side electrode is shown for clarity). The abducens nucleus was searched by stereotaxic approach and followed by the recording of the antidromic field potential evoked after electrical stimulation (50 µs, < 0.1 mA) of the ipsilateral (left) VIth nerve (Fig. 1, St.). Units that were isolated in the abducens nucleus but that were neither antidromically activated nor collided after electrical stimulation to the VIth nerve were considered as ABD Ints. Thus, ABD Ints were recorded in the left abducens nucleus (Fig. 1, Rec., in purple). Recordings of ATD neurons were carried out in the right ventrolateral vestibular nucleus (Fig. 1, Rec., in green), using the abducens nucleus as a reference (see below). Medial rectus motoneurons from our previous publication (Hernández et al. 2017) were recorded on the right side (Fig. 1, Rec., in orange) and were antidromically activated from the ipsilateral IIIrd nerve (Fig. 1, St.). For more details about medial rectus motoneuron recordings, see Hernández et al. (2017). Thus, recordings were carried out reproducing the anatomical connections: left ABD Ints and right ATD neurons project onto right medial rectus motoneurons (Fig. 1). Multistranded wire made of Teflon-isolated stainless steel was used to construct eye coils that were sutured to the sclera of both eyes. A craniotomy (5 × 5 mm) was carried out in the supraoccipital bony crest to allow the passage of micropipettes toward the brainstem. A head-restraining system was also constructed with dental acrylic for immobilization purposes during recordings. Pre- and postoperative care was provided throughout the experiment, as required.

### Chronic extracellular recordings

Recordings followed our previously published methods (Calvo et al. 2020, 2022). Recording sessions started after a postoperative recovery of no less than 10 days. The animal was placed in a cloth bag and wrapped with elastic bandages to limit its body movements. Then, the animal was positioned in a cushioned methacrylate box with its head immobilized. Special care was taken to keep the animal in a comfortable position during recording sessions. We used the scleral search-coil technique described by Fuchs and Robinson (1966) to record eye movements. Neuronal activity was recorded using glass micropipettes filled with 2 M NaCl. The different recording locations (ABD and LVN) were searched through a transcerebellar stereotaxic approach using a three-axis micromanipulator to drive the micropipette.

The electrophysiological center of the abducens nucleus was located at the coordinates where a maximum antidromic field potential was attained. Then the new recording location was moved between 2 and 2.5 mm lateral and 1–1.5 mm dorsal to the abducens nucleus, for the recording of ATD neurons (lateral vestibular nucleus, ventral division), according to Berman (1968). We considered ATD neurons those isolated within these coordinates that responded as a type I neuron during horizontal head rotation (Gernandt 1949), that is, its discharge increased for head rotations towards the ipsilateral side of the recording. Single-unit extracellular action potentials were recorded during both spontaneous and vestibularly induced eye movements. For spontaneous eye movements we simply allowed the animal to look around scanning for the different objects of the laboratory, that is, animals were not trained to look for a particular spot of light. Vestibular stimulation in the horizontal plane was delivered by a motorized turntable to generate sinusoidal rotations of the table at a frequency of 0.125 Hz and an amplitude range between ± 20 and ± 30 degrees zero to peak. Neuronal firing was amplified and filtered (10 Hz–10 kHz).

### Data storage and analysis

The recording of horizontal eye position of both eyes and the simultaneous neuronal activity were digitally stored in a computer (Power 1401, Cambridge Electronic Design, Cambridge, UK). Matlab 7.5 computer programs were used for selecting epochs of data containing the instantaneous firing frequency (the reciprocal of the interspike intervals) and the eye and head position. Eye and head velocity were digitally differentiated.

The firing rate of extraocular motoneurons contains a static component related to eye position and a dynamic component related to eye velocity. Thus, the equation described for the discharge of these cells corresponds to FR = F_0_ + k · EP + r · EV (Calvo et al. 2022, 2023; Davis-López de Carrizosa et al. 2011; Robinson 1970). In this equation FR is the firing rate of the neuron (in spikes/s), F_0_ is the firing rate when the eye is centered in the orbit (i.e., looking ahead), k represents the unit eye position sensitivity (in spikes/s/degree), EP is eye position (in degrees), r is the unit eye velocity sensitivity (in spikes/s/degree/s) and EV is eye velocity (in degrees/s) (Calvo et al. 2022, 2023; Davis-López de Carrizosa et al. 2011). For spontaneous eye movements, neuronal eye position and velocity sensitivities were named *k*_s_ and *r*_s_, and for vestibular eye movements, they were designed as *k*_v_ and *r*_v_. Eye-related parameters for ATD neurons, ABD Ints, and medial rectus motoneurons were calculated with respect to their respective ipsilateral eye.

During ocular fixations, since eye velocity is zero, the equation simplifies to FR = F_0_ + *k*_s_ · EP. We calculated the *k*_s_ coefficient by linear regression fitting so that the slope of the linear regression thus obtained represents the neuronal eye position sensitivity for ocular fixations (i.e., *k*_s_) (Calvo et al. 2022, 2023; Davis-López de Carrizosa et al. 2011). We also calculated the eye position threshold for neuronal recruitment into activity as the eye position value at which FR = 0, therefore, from the above equation, threshold was obtained for each neuron as − F_0_/*k*_s_ (in degrees). During saccades, we correlated firing rate, after subtracting the eye position component (*k*_s_ · EP), with eye velocity, so that the above equation changes to FR− *k*_s_ · EP = F_0_ + *r*_s_ · EV. Therefore, the slope of the linear regression is the neuronal eye velocity sensitivity obtained for spontaneous saccades (*r*_s_).

During vestibularly induced eye movements, the equation used was FR = F_0_ + *k*_v_ · EP + *r*_v_ · EV. We selected between cursors the slow phases of the nystagmus and used the multiple regression analysis to obtain the neuronal sensitivities *k*_v_ and *r*_v_.

### Statistics

Comparisons between groups were performed using the one-way ANOVA test, in all cases at an overall level of significance of *p* < 0.05. ANOVA tests were followed by post hoc pairwise multiple comparisons, by means of the program SigmaPlot version 11 (Systat Software, Inc., San Jose, CA, USA). All regression equations obtained from the fit of firing rate with eye position and eye velocity were significant (*p* < 0.05). The effect size was indicated by Cohen’s d (*d*). Quantitative data are represented with box-and-whisker plots showing the median, 25th (Q1), and 75th (Q3) quartiles, with 90th and 10th percentiles as error bars. All data points are superimposed in the graphs.

## Results

### Discharge pattern of medial rectus motoneurons and their afferent sources LVN and ABD Ints during spontaneous eye movements

**Fig. 2:**
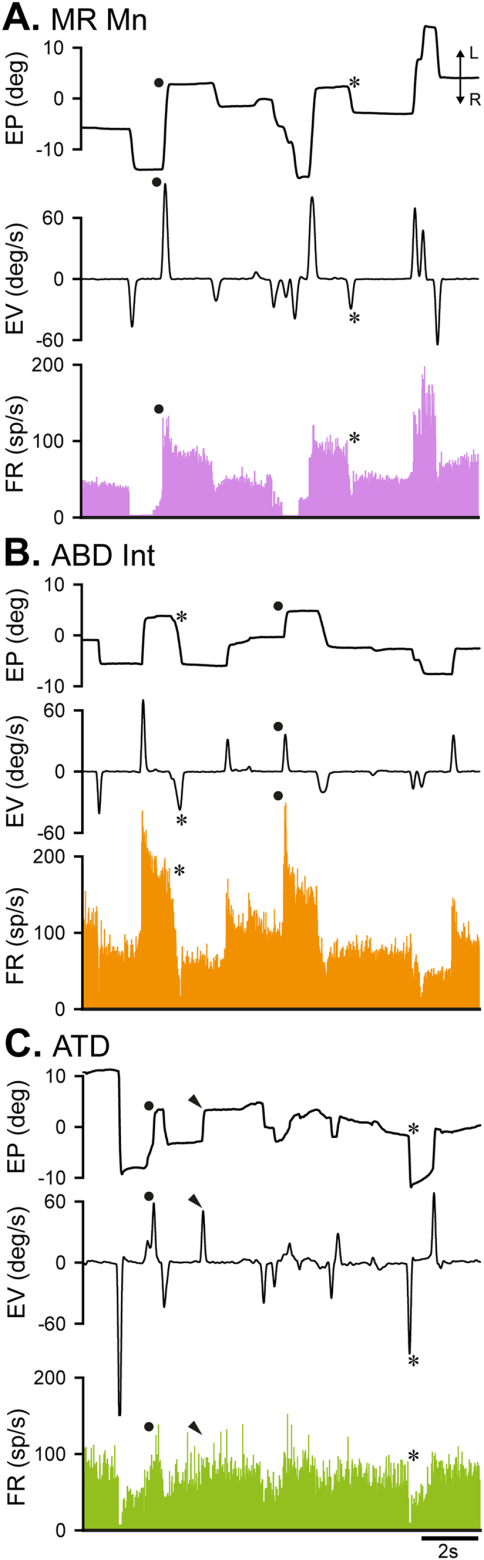
Firing of MR motoneurons, ABD Ints, and ATD neurons, during spontaneous eye movements. The figure shows examples of the firing rate (FR, in spikes/s) during spontaneous eye movements of the three neuronal types studied: **A** MR motoneurons (Mns), **B** internuclear neurons (Ints) of the ABD, and **C** vestibular neurons of the ATD. L and R next to the double arrow in A stands for leftward and rightward eye movements, respectively (for **A**–**C**). MR motoneurons and ATD neurons were recorded in the right side, and ABD Ints in the left side. The horizontal eye position (EP, eye position, in degrees) and velocity (EV, eye velocity, in degrees/s) are shown for the eye ipsilateral to the recording site in (**A**–**C**). Note that the discharge of the three neurons increases with eye movements toward the left side, although the correlation between FR and EP and EV can be better appreciated in the ABD Int (**B**) and the MR Mn (**A**) as compared to the ATD neuron (**C**). Solid dots point to burst-like increases in FR during on-directed saccades, asterisks indicate an abrupt decay in FR for off-directed saccades, and arrowheads show an example of absence of FR response during saccades in the ATD neuron (**C**)

Medial rectus motoneurons showed a phasic-tonic firing profile that was found linearly correlated to eye position and eye velocity. During fixations, they increased their firing rate for eye positions located towards the on direction, which was contralateral to the recording side (i.e., the nasal direction of the ipsilateral eye). Since recordings were carried out in the right oculomotor nucleus, this means that discharge frequency was higher when gaze was directed toward the left side (Fig. 2A). In addition, medial rectus motoneurons displayed a phasic component which was present for rapid eye movements. They exhibited a brisk burst of spikes for saccades in the on direction (Fig. 2A, solid dots) and an abrupt decay in firing or a pause for saccades in the off direction (Fig. 2A, asterisks). ABD Ints exhibited a tonic-phasic discharge pattern that was similar to that of medial rectus motoneurons, except that their on direction was the opposite, i.e., towards the ipsilateral side of the recording (i.e., the temporal direction of the ipsilateral eye). As recordings were obtained in the left abducens nucleus, their discharge increased for eye movements towards the left side. Nevertheless, ABD Ints carry appropriate information to medial rectus motoneurons, since their axons cross the midline and terminate via excitatory synapses on these motoneurons located in the contralateral oculomotor nucleus (Fig. 1; Highstein and Baker 1978). Thus, given that medial rectus motoneurons were recorded in the right oculomotor nucleus and ABD Ints in the left abducens nucleus, the on-direction of both cell types was the same, i.e., toward the left (Fig. 2B). In general, it was observed that ABD Ints fired at higher tonic-phasic rates than medial rectus motoneurons, in particular, the firing bursts for on-directed saccades reached higher frequencies (Fig. 2B, solid dots). Similar to medial rectus motoneurons, ABD Ints rapidly decreased or stopped firing during off-directed saccades (Fig. 2B, asterisks).

During spontaneous eye movements, the discharge of ATD neurons differed somewhat from that of motoneurons and ABD Ints. Thus, as can be appreciated in Fig. 2C, although they also modulated in relation to eye movements, their degree of correlation with eye position was less conspicuous. They increased their discharge rate as eye positions were fixed more to the contralateral side (left) of the recording (right) site, as medial rectus motoneurons did. Since ATD neurons send excitatory projections to the ipsilateral oculomotor nucleus (Baker and Highstein 1978), the signals conveyed by these premotor cells were appropriate for driving medial rectus motoneurons. Therefore, the three neuronal types recorded as illustrated in Fig. 1 presented eye movements to the left as the on-direction. The behavior of ATD neurons also showed a phasic component. During on-directed saccades, they discharged a burst of spikes, which was, however, of low frequency (Fig. 2C, solid dots), and on occasions, it was not present (Fig. 2C, arrowheads). For off-saccades, ATD neurons usually decreased their firing rate (Fig. 2C, asterisks), and rarely they showed no change in their discharge activity or this was not consistent. Thus, their phasic activity was present (Fig. 2C), but it was not as precise as that of medial rectus motoneurons (Fig. 2A) and ABD Ints (Fig. 2B).

### Quantitative comparison of the signals displayed during spontaneous eye movements in ATD neurons, ABD Ints and their target medial rectus motoneurons

A comparison of eye-related parameters between the three neuronal types was carried out during spontaneous eye movements. Although our principal aim was the comparison between ATD neurons and ABD Ints, we also included the discharge properties of medial rectus motoneurons to discern the degree of similarity in the firing activity of both inputs with respect to their target motoneurons. The number of neurons analyzed during spontaneous eye movements was *n* = 25 medial rectus motoneurons, *n* = 14 ABD Ints, and *n* = 13 ATD neurons.

**Fig. 3:**
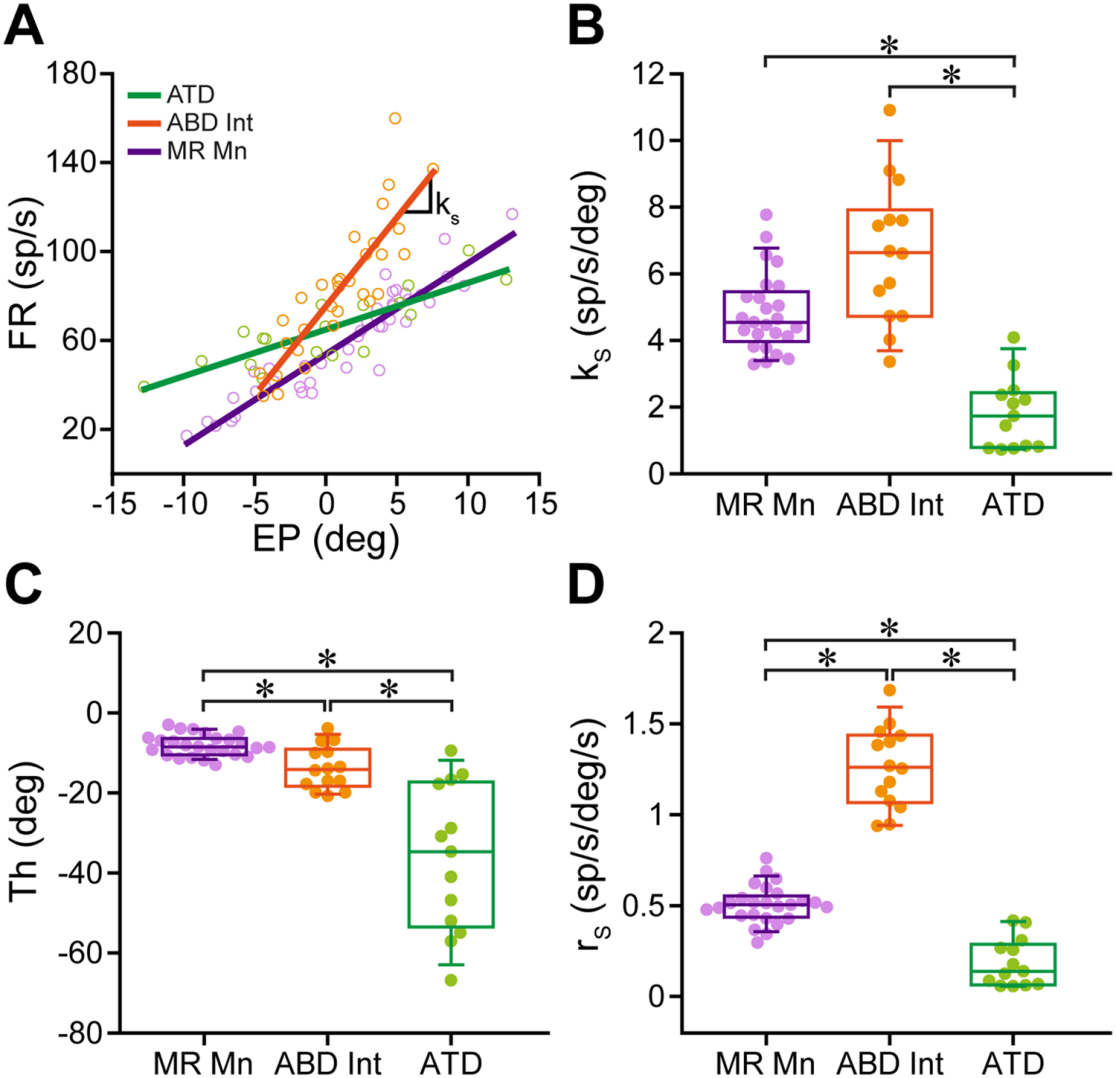
Quantitative comparison of eye-related sensitivities obtained during spontaneous fixations and saccades between MR motoneurons and their two major pontine inputs. **A** Correlation between firing rate (FR, in spikes/s) and eye position (EP, in degrees) was carried out by linear regression analysis. The slope of the regression line thus obtained represents the neuronal eye position sensitivity (*k*_s_, in spikes/s/degree). The three lines correspond to the neurons shown in Fig. 2, whose *k*_s_ values were 4.1, 7.9, and 2.1 spikes/s/degree, for the MR Mn of Fig. 2A, the ABD Int of Fig. 2B, and the ATD neuron of Fig. 2C, respectively. **B** Box-and-whisker plot showing eye position sensitivity during eye fixations (*k*_s_, in spikes/s/degree) between MR Mns, ABD Ints, and ATD neurons. One-way ANOVA test revealed significant differences (*p* ≤ 0.001) between groups. Pairwise multiple comparisons (Dunn’s method) demonstrated that ABD Ints and MR Mns showed significantly higher *k*_s_ values than ATD neurons (*p* < 0.05 for both cases; asterisks). **C** Same as **B** but for the eye position threshold at which the neuron was recruited into activity (Th, in degrees). One-way ANOVA test revealed significant differences (*p* ≤ 0.001) between groups. All pairwise multiple comparisons (Dunn’s method) were significantly different (*p* < 0.05 for the three cases; asterisks). ATD neurons showed the lowest threshold and MR Mns the highest threshold. **D** Same as **B** but for eye velocity sensitivity during saccades (*r*_s_, in spikes/s/degree/s). Significant differences were present between the three groups (one-way ANOVA,* p* ≤ 0.001). When pairwise multiple comparisons were carried out (Dunn’s method) all of them resulted in significant differences (*p* < 0.05). ABD Ints showed the highest *r*_s_ values, while ATD neurons presented the lowest. For **B**–**D**, *n* = 25 MR Mns, 14 ABD Ints, and 13 ATD neurons

During fixations, neuronal eye position sensitivity (*k*_s_) was calculated as the slope of the regression line between firing rate and eye position. The result of this analysis for the three neuronal populations illustrated in Fig. 2 is shown in Fig. 3A. It can be appreciated that the slopes, and therefore *k*_s_ values, of the ABD Int and the medial rectus motoneuron, were higher than that of the ATD neuron.

When neuronal eye position sensitivities (*k*_s_) were compared between the three neuronal populations, it was found that *k*_s_ values of ATD neurons were significantly lower than those of both ABD Ints and medial rectus motoneurons (Fig. 3B; one-way ANOVA on ranks followed by Dunn’s method for post hoc comparison*s*, *p* ≤ 0.001, *H* = 31.470, Cohen’s *d* = 2.287). Mean ± SEM *k*_s_ values were 4.81 ± 0.24, 6.62 ± 0.57, and 1.81 ± 0.3 spikes/s/degree, for medial rectus motoneurons, ABD Ints, and ATD neurons, respectively. Moreover, *k*_s_ values of ABD Ints were similar to those of motoneurons (*p* > 0.05) whereas those of ATD neurons were significantly lower (*p* < 0.05) (Fig. 3B). It should be noted that, however, the statistical comparison of the coefficients of determination (*R*^2^) for the rate-position regression equations between the three populations showed no statistical difference (ANOVA on ranks, *p* = 0.158, *H* = 3.696), indicating that the *k*_*s*_ signal was present in the two premotor inputs, as well as in the motoneurons.

The eye position threshold for recruitment into activity was also calculated from the regression equations and compared between the three groups. Interestingly, in this case, there was a significant difference between all neuronal populations, so that ATD neurons showed the lowest threshold and medial rectus motoneurons the highest (Fig. 3C; one-way ANOVA on ranks followed by Dunn’s multiple comparisons, *p* ≤ 0.001, H = 28.013, *d* = 2.001). Therefore, the recruitment threshold of the two afferent populations (ATD neurons and ABD Ints) was lower than that of the motoneurons they terminate on. In turn, the threshold of ATD neurons was significantly (*p* < 0.05) lower than that of ABD Ints, that is, they were recruited into activity at more negative (off-directed) eye positions in the orbit. We would like to highlight that the results in threshold were quite striking, particularly due to the extremely low threshold of ATD neurons as compared to ABD Ints and medial rectus motoneurons (mean ± SEM were: − 36.37 ± 5.11 degrees, − 13.71 ± 1.46, and − 8.17 ± 0.54, respectively).

During saccades, the neuronal eye velocity sensitivities (*r*_*s*_) obtained for the three populations reflected the qualitative observations described above from their respective firing pattern. Thus, ABD Ints showed the highest *r*_s_ values (1.26 ± 0.06 spikes/s/degree/s), and ATD neurons had the lowest *r*_s_ values (0.19 ± 0.04 spikes/s/degree/s). Medial rectus motoneuronal data were in between (0.50 ± 0.02 spikes/s/degree/s). The differences between the three groups reached statistical significance (Fig. 3D; one-way ANOVA on ranks followed by Dunn’s multiple comparisons, *p* ≤ 0.001, *H* = 42.109, *d* = 3.673). The saccadic signal of ATD neurons was weak and not always consistent. This yielded low correlations of determination of the rate-velocity regression equations for ATD neurons, which were significantly lower than those obtained for both ABD Ints and motoneurons (one-way ANOVA followed by Holm–Sidak’s multiple comparisons, *F*(2,49) = 60.517, *p* ≤ 0.001). It should be highlighted that with respect to all eye-related parameters calculated in the present work, only eye velocity sensitivity for spontaneous saccades (*r*_s_) produced significantly lower coefficients of determination in ATD neurons compared to ABD Ints and medial rectus motoneurons. In all other parameters, the three groups of neurons showed similar coefficients of determination. Therefore, the saccadic signal in ATD neurons was not as accurate as that of the other main input to the medial rectus motoneurons, the ABD Ints.

### Discharge pattern of medial rectus motoneurons, ABD Ints, and ATD neurons during vestibular eye movements

**Fig. 4:**
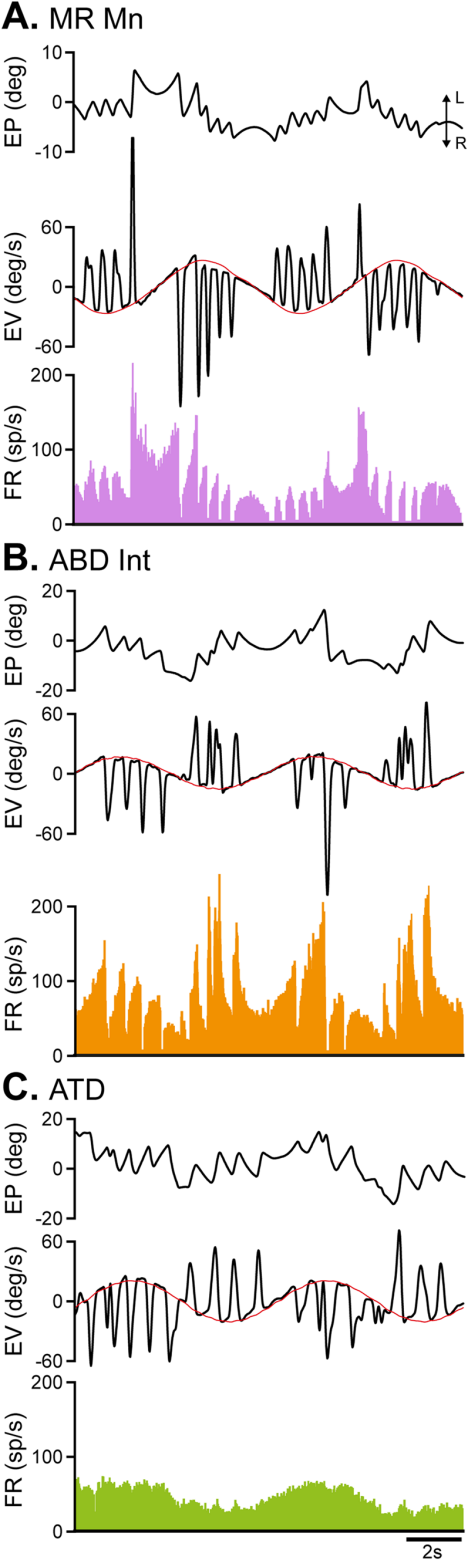
Behavior of MR Mns, ABD Ints, and ATD neurons during vestibular eye movements. Traces in **A**–**C** illustrate from top to bottom: eye position (EP, in degrees), eye velocity (EV, in degrees/s), and firing rate (FR, in spikes/s). The red trace in EV represents head velocity, which is shown inverted for clarity. L and R next to the double arrow in **A** indicate leftward and rightward eye movements, respectively (for **A**–**C**). MR motoneurons and ATD neurons were recorded in the right side, and ABD Ints in the left side. Note that in the three neuronal types (**A**–**C**), on-directed eye movements were those toward the left. The firing pattern of MR Mns (**A**) and ABD Ints (**B**) during vestibularly induced eye movements was similar. Thus, their discharge increased for slow and fast phases of the nystagmus in the on direction and decreased for slow and fast phases in the opposite direction. However, ATD neurons clearly modulated during the slow phases of the vestibulo-ocular reflex but lacked any signal related to fast phases (**C**). Note also that whereas ATD neurons and MR Mns showed type I response during vestibular eye movements (i.e., their discharge increased for head rotations toward the ipsilateral side of the recording), ABD Ints showed type II response (i.e., firing increased for head rotations toward the contralateral recording side)

During vestibularly induced eye movements the firing profile of medial rectus motoneurons and that of ABD Ints was similar (Fig. 4A, B). The two neuronal types modulated during both the slow and the fast phases of the nystagmus. However, whereas the discharge of medial rectus motoneurons increased for head rotations towards the same side of the recording (type I response; Gernandt 1949), ABD Ints increased their firing for head rotations towards the opposite side (type II response; Gernandt 1949). As mentioned above, as ABD Ints were recorded in the left side and medial rectus motoneurons in the right side, then both neuronal types increased their discharge for rightward head rotations resulting in eye movements to the left. As can be observed in Fig. 4A, B, although they showed the same discharge pattern, ABD Ints reached, in most cases, higher firing frequencies than medial rectus motoneurons during vestibular eye movements.

The response of ATD neurons during vestibularly induced eye movements was type I since their firing increased for head rotations towards the ipsilateral side of the recording (Gernandt 1949; Markham et al. 1986). During vestibular eye movements, their firing pattern differed from that of motoneurons and ABD Ints (Fig. 4C). They modulated sinusoidally (as the stimulus) without showing bursts or decreases in activity for the on- and off-directed fast phases of the nystagmus, respectively, in marked contrast to motoneurons and ABD Ints (Fig. 4A, B). This behavior likely corresponds to the head velocity signal described by Reisine and Highstein (1979).

### Quantitative comparison of ATD neurons and ABD Ints signals during vestibular eye movements in relation to medial rectus motoneurons

**Fig. 5:**
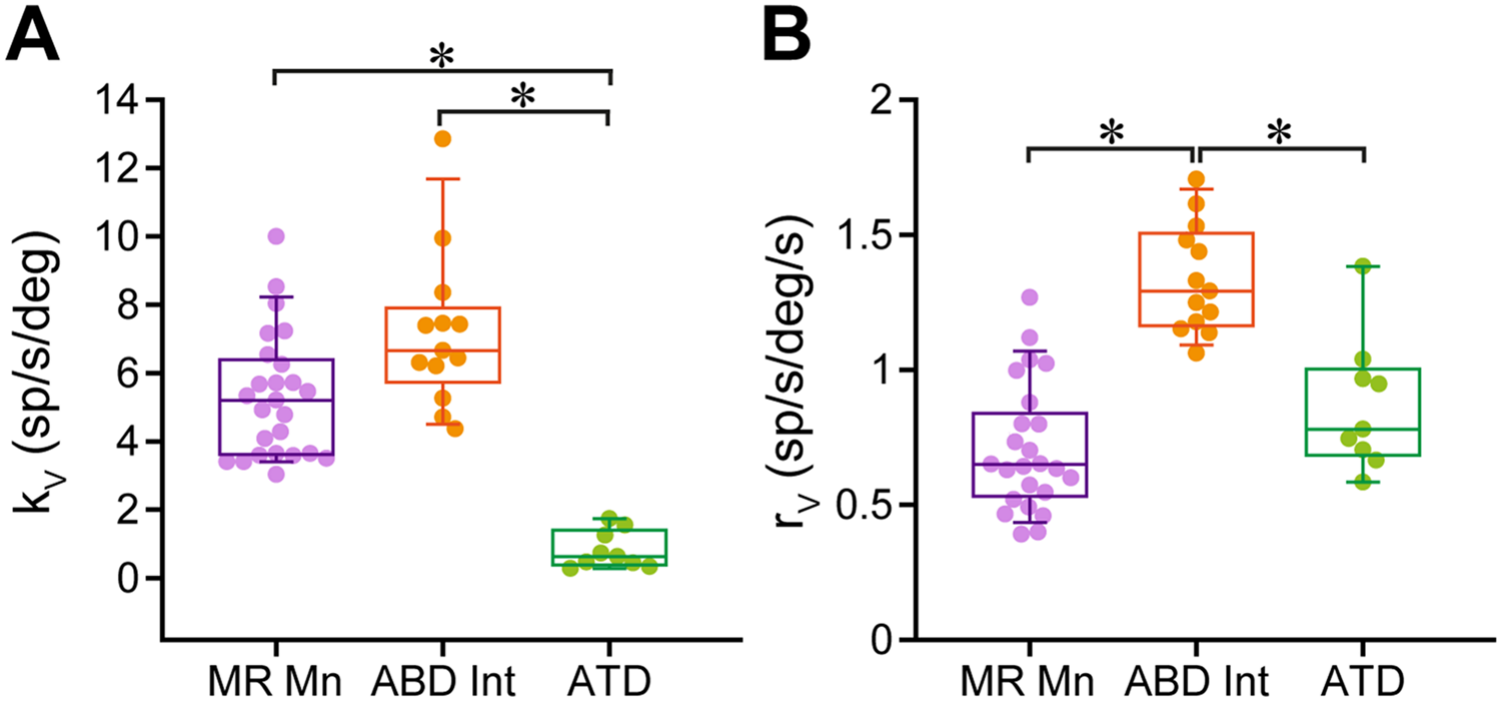
Eye-related parameters of MR Mns, ABD Ints, and ATD neurons during vestibular eye movements. **A** Box-and-whisker plot illustrating the values of neuronal eye position sensitivity during the slow phases of the vestibular nystagmus (*k*_v_, in spikes/s/degree) for MR Mns, ABD Ints, and ATD neurons. One-way ANOVA on ranks detected significant differences between groups (*p* ≤ 0.001). Post-hoc pairwise multiple comparisons (Dunn’s method) revealed that MR Mns and ABD Ints showed significantly (*p* < 0.05, asterisks) higher *k*_v_ values than ATD neurons. **B** Box-and-whisker plot showing the values of neuronal eye velocity sensitivity during the slow phases of the vestibulo-ocular reflex *r*_v_ (in spikes/s/degree/s). One-way ANOVA on ranks revealed that there were significant differences between groups (*p* ≤ 0.001). When pairwise multiple comparisons were performed (Dunn’s method), we obtained that ABD Ints showed significantly higher *r*_v_ values than both ATD neurons (*p* < 0.05) and MR Mns (*p* < 0.05). The number of neurons analyzed in **A** and **B** was 25 MR Mns, 13 ABD Ints, and 9 ATD neurons

Eye position and eye velocity sensitivities were calculated during vestibular eye movements (namely *k*_v_ and *r*_v_, respectively) selecting the slow phases of the nystagmus and using multiple regression analysis. ABD Ints showed higher *k*_v_ values than ATD neurons (Fig. 5A; one-way ANOVA on ranks followed by Dunn’s multiple comparisons, *p* ≤ 0.001, *H* = 25.816, *d* = 1.838). Mean ± SEM data were 7.18 ± 0.63 and 0.83 ± 0.18 spikes/s/degree, respectively. When compared with medial rectus motoneurons (5.31 ± 0.37 spikes/s/degree), ABD Ints showed similar *k*_v_ (*p* > 0.05), whereas *k*_v_ in ATD neurons was significantly lower than in the motoneurons (*p* < 0.05) (Fig. 5A).

Regarding eye velocity sensitivity (*r*_v_), ABD Ints also exhibited higher values (1.37 ± 0.06 spikes/s/degree/s) than ATD neurons (0.87 ± 0.08 spikes/s/degree/s) (Fig. 5B; one-way ANOVA on ranks followed by Dunn’s method for post hoc comparisons, *p* ≤ 0.001, *H* = 26.054, *d* = 1.856). However, in this case, eye velocity sensitivity of ATD neurons during vestibular eye movements (*r*_v_) was similar (*p* > 0.05) to that of motoneurons (0.71 ± 0.05 spikes/s/degree/s), whereas ABD Ints showed significantly higher *r*_v_ values than motoneurons and ATD neurons (*p* < 0.05) (Fig. 5B).

## Discussion

### A comparison of eye-movement signals between ABD interneurons and ATD neurons

The results of the present work have demonstrated that ATD neurons exhibit eye position and eye velocity sensitivities during both spontaneous and vestibular eye movements. Nevertheless, these sensitivities were significantly lower than those of ABD Ints. During spontaneous eye movements, the eye position signal of ATD neurons was weak, and their response during saccades was not as consistent as that of ABD Ints. On the other hand, during vestibular eye movements, ATD neurons clearly modulated with eye movements, increasing their firing for head rotation towards the ipsilateral direction (type I response) but lacked the phasic component during the fast phases of the vestibular nystagmus. ABD Ints exhibited a type II response and modulated during both slow and fast phases of the nystagmus. Eye position and eye velocity sensitivities for both spontaneous and vestibular eye movements (*k*_s_, *r*_*s*_, *k*_v_, and *r*_v_) were significantly higher in ABD Ints as compared to ATD neurons, likely indicating that ABD Ints constitute the major input driving the medial rectus motoneuron discharge (Delgado-Garcia et al. 1986; Hernández et al. 2017; Highstein and Baker 1978).

However, it should be noted that the coefficients of determination of the rate-position and rate-velocity plots in ATD neurons were similar to those of ABD Ints for spontaneous and vestibular eye movements. The only noticeable difference was noted in eye velocity sensitivity during spontaneous saccades (*r*_s_) because coefficients of determination for ATD neurons were significantly lower. This was evident in the firing activity of ATD neurons during saccades, which was not as accurate as that of ABD Ints, and on occasions, these vestibular neurons lacked any response or displayed an inappropriate signal during saccades.

As stated above, previous recordings of ATD neurons revealed that these neurons convey a head velocity and a weak eye position signal to medial rectus motoneurons (Reisine and Highstein 1979; Reisine et al. 1981). Since ABD Ints discharge in a tonic-phasic fashion during spontaneous and vestibular eye movements (Delgado-Garcia et al. 1986; Fuchs et al. 1988), information coming from ATD neurons has been suggested as redundant (Furuya and Markham 1981). However, other authors have claimed that, as occurs for the other extraoculomotor neurons, a monosynaptic head velocity signal from ATD neurons may provide medial rectus motoneurons with a head velocity signal, which would be required to overcome the visco-elastic forces of the ocular tissues, and effectively produce a vestibulo-ocular reflex in which the head velocity would be effectively compensated by the corresponding eye velocity (Reisine and Highstein 1979).

### Differences in the projection of ABD interneurons and ATD neurons onto medial rectus motoneurons

Intracellular recordings of medial rectus motoneurons carried out in acute cats following electrical stimulation applied to the ipsilateral vestibular nerve have shown high amplitude disynaptic EPSPs originating from ATD neurons. These EPSPs reverse with the injection of small depolarizing currents, indicating a somatic and/or proximal dendritic location of this input (Baker and Highstein 1978). The electrical stimulation to ABD Ints produces also large (monosynaptic) EPSPs on medial rectus motoneurons, but the reversal of these synaptic potentials requires currents of higher intensity indicative of a more distal location in the somatodendritic compartment (Highstein and Baker 1978). These results were later confirmed at the electron microscopy level by the anterograde labeling of each afferent population and the observation of their respective labeled nerve endings on medial rectus motoneurons (Nguyen et al. 1999). That study demonstrated that the two excitatory inputs differ in their somato-dendritic location: the majority of ATD synaptic endings contact proximal dendrites or somata, whereas most abducens synaptic boutons terminate on distal dendrites (Nguyen et al. 1999). In contrast, by means of anterograde labeling, the vestibular projection has been shown to be much less abundant on the medial rectus motoneuron subgroup in the oculomotor nucleus, as compared with the massive projection of afferent terminals arising from the ABD Ints (Carpenter and Carleton 1983; Hernández et al. 2017).

### Possible roles of ATD input onto medial rectus motoneurons

An outstanding result of the present work was the very markedly low eye position recruitment threshold present in ATD neurons compared with ABD Ints (as well as with medial rectus motoneurons). This finding indicates that ATD neurons may maintain medial rectus motoneurons at a low level of tonic excitatory influence. Therefore, the ATD input may facilitate the recruitment of motoneuron firing by the more distal projection from the abducens internuclear pathway. In this respect, we suggest that the very low threshold of ATD neurons might play a relevant role by facilitating the transmission of signals from ABD Ints onto medial rectus motoneurons. Another role suggested in the literature (Chen-Huang and McCrea 1998) is the important action of ATD neurons during the cancellation of the rotational vestibulo-ocular reflex by means of targets located at different distance from the subject. Thus, as the visual target is positioned closer to the subject, the eye movement required to stabilize the image increases dramatically. A closer visual target increases the angle of convergence, and it was found in the squirrel monkey that ATD firing during the VOR increases accordingly for closer viewing (Chen-Huang and McCrea 1998).

According to the present data, the ATD signals, compared to those found in the ABD Int population, seem to be weak by themselves for the induction of high firing rates in medial rectus motoneurons, which are required for nasally directed eye movements of certain amplitude. Indeed, MLF lesions rostral to the abducens nucleus, which interrupt the abducens internuclear pathway, lead to deficits in conjugate eye movements in the horizontal plane, but preserves convergent eye movements (the convergent signal arises from a different afferent, likely the midbrain near response cells, Zhang et al. 1992). Thus, after MLF lesion, the ipsilateral eye shows an inability to adduct across the midline, and eye movements are restricted to the ipsilateral oculomotor hemifield (de la Cruz et al. 2000). In the clinics, these motor deficits are identified as the syndrome of internuclear ophthalmoplegia (Carpenter and McMasters 1963; Christoff et al. 1960; Lee et al. 2022; Pola and Robinson 1976) and have also been experimentally induced in cats and monkeys by the lesion or inactivation of the MLF (de la Cruz et al. 2000; Evinger et al. 1977; Gamlin et al. 1989). The fact that the lesion of the MLF, through which ABD Int axons traverse, leads to the incapacity of the ipsilateral eye to cross midline towards the contralateral hemifield, even though the ATD pathway is intact, implies that ATD neurons are not able to activate medial rectus motoneurons enough to cause the eye to cross the midline. In patients suffering from unilateral or bilateral MLF lesions, there is partial preservation of the horizontal vestibulo-ocular reflex, indicating a role for ATD neurons in mediating, at least in part, horizontal vestibular eye movements (Aw et al. 2017). However, those patients are unable to perform adducting horizontal saccades during the vestibulo-ocular reflex (Aw et al. 2017), in congruence with the present data, showing absence of saccadic responses in ATD neurons during the fast phases of the vestibular nystagmus.

### Effects of MLF and ATD section on the activity of medial rectus motoneurons

The selective unilateral section of the ATD in cats produces oculomotor deficits in the ipsilateral eye, such as a reduction of range during spontaneous eye movements and a decrease in the gain of the vestibulo-ocular reflex, although all those alterations recover over time, due to compensation from the intact MLF pathway. In addition, the section of the ATD leads to a reduction in eye position and eye velocity sensitivities of medial rectus motoneurons recorded under alert conditions, during both spontaneous and vestibular eye movements (Hernández et al. 2017). Those results imply that vestibular neurons projecting to medial rectus motoneurons contribute by their synaptic action to generate eye-related signals in the discharge pattern of these motoneurons. However, the reduction in oculomotor signals displayed by medial rectus motoneurons after ATD transection are short-lasting (5–7 days), due to axonal sprouting of the intact ABD Int pathway that compensates for all the alterations induced by the ATD section in a short time. In contrast, when the MLF is sectioned leaving intact the ATD, changes observed in eye movements and medial rectus motoneuron discharge are of higher magnitude and of longer duration. In this case, compensation for the loss of MLF input by intact ATD axons is only partial (Hernández et al. 2017). Accordingly, ABD Ints play a more powerful synaptic influence on the firing activity of medial rectus motoneurons under alert conditions than ATD neurons, and are endowed with a larger degree of plastic mechanisms such as axonal sprouting after lesion. On the other hand, ATD neurons are not a redundant input onto medial rectus motoneurons, as their loss disturbs eye movements and motoneuron discharge.

### Comparing the discharge of medial rectus motoneurons with that of their two main inputs

A comparison of eye-related parameters of both inputs with those of medial rectus motoneurons yielded different results. ABD Ints showed similar eye position sensitivity to medial rectus motoneurons during both spontaneous and vestibular eye movements (i.e., *k*_s_ and *k*_v_). However, eye velocity sensitivities for spontaneous saccades (*r*_s_) and vestibular-induced eye movements (*r*_v_) were higher in ABD Ints than in motoneurons. This likely reflects a greater synaptic influence of ABD Ints when the medial rectus muscle requires a stronger force. On the other hand, ATD neurons showed lower eye position (*k*_s_) and velocity (*r*_s_) sensitivities than medial rectus motoneurons during spontaneous movements. However, during vestibular eye movements, although ATD neurons presented a lower eye position sensitivity (*k*_v_), the eye velocity sensitivity (*r*_v_) was similar to that of motoneurons. Thus, this may be the major signal conveyed by ATD neurons on medial rectus motoneurons, and is in consonance with the head velocity signal previously reported for ATD neurons (Reisine and Highstein 1979; Reisine et al. 1981).

In summary, it might be concluded that the signals encoded by ATD neurons and ABD Ints act together on the population of medial rectus motoneurons to finally lead to the adequate functioning of the horizontal vestibulo-ocular reflex and the conjugation of eye movements in the horizontal plane. The lower threshold of ATD neurons might favor the activation of motoneurons by the ABD Int pathway. Nevertheless, both inputs are required for the normal operation mode of medial rectus motoneurons under physiological conditions.

## Data Availability

Data are available upon kind request.
